# Human Ovarian Granulosa Cells Isolated during an IVF Procedure Exhibit Differential Expression of Genes Regulating Cell Division and Mitotic Spindle Formation

**DOI:** 10.3390/jcm8122026

**Published:** 2019-11-20

**Authors:** Maciej Brązert, Wiesława Kranc, Błażej Chermuła, Katarzyna Kowalska, Maurycy Jankowski, Piotr Celichowski, Michal Jeseta, Hanna Piotrowska-Kempisty, Leszek Pawelczyk, Maciej Zabel, Paul Mozdziak, Bartosz Kempisty

**Affiliations:** 1 Division of Infertility and Reproductive Endocrinology, Department of Gynecology, Obstetrics and Gynecological Oncology, Poznan University of Medical Sciences, 33 Polna St., 60-535 Poznań, Poland; maciejbrazert@ump.edu.pl (M.B.); blazej.chermula@wp.pl (B.C.); pawelczyk.leszek@ump.edu.pl (L.P.); 2 Department of Anatomy, Poznan University of Medical Sciences, 6 Święcickiego St., 60-781 Poznań, Poland; wkranc@ump.edu.pl (W.K.); mjankowski@ump.edu.pl (M.J.); 3 Department of Histology and Embryology, Poznan University of Medical Sciences, 6 Święcickiego St., 60-781 Poznań, Poland; kkowalsk@ump.edu.pl (K.K.); p.celichowski@gmail.com (P.C.); 4 Department of Obstetrics and Gynecology, University Hospital and Masaryk University, 20 Jihlavská St., 625 00 Brno, Czech Republic; jeseta@gmail.com; 5 Department of Toxicology, Poznan University of Medical Sciences, 30 Dojazd St., 60-631 Poznań, Poland; hpiotrow@ump.edu.pl; 6 Department of Histology and Embryology, Wroclaw Medical University, Chałubińskiego St., 50-368 Wrocław, Poland; m.zabel@wlnz.uz.zgora.pl; 7 Division of Anatomy and Histology, University of Zielona Gora, 28 Zyty St., 65-046 Zielona Góra, Poland; 8 Physiology Graduate Program, North Carolina State University, Campus Box 7608, Raleigh, NC 27695-7608, USA; pemozdzi@ncsu.edu

**Keywords:** ovarian granulosa, human, in vitro, cell division

## Abstract

Granulosa cells (GCs) are a population of somatic cells whose role after ovulation is progesterone production. GCs were collected from patients undergoing controlled ovarian stimulation during an in vitro fertilization procedure, and they were maintained for 1, 7, 15, and 30 days of in vitro primary culture before collection for further gene expression analysis. A study of genes involved in the biological processes of interest was carried out using expression microarrays. To validate the obtained results, Reverse Transcription quantitative Polymerase Chain Reaction (RT-qPCR) was performed. The direction of changes in the expression of the selected genes was confirmed in most of the examples. Six ontological groups (“cell cycle arrest”, “cell cycle process”, “mitotic spindle organization”, “mitotic spindle assembly checkpoint”, “mitotic spindle assembly”, and “mitotic spindle checkpoint”) were analyzed in this study. The results of the microarrays obtained by us allowed us to identify two groups of genes whose expressions were the most upregulated (*FAM64A, ANLN, TOP2A, CTGF, CEP55, BIRC5, PRC1, DLGAP5, GAS6,* and *NDRG1*) and the most downregulated (*EREG, PID1, INHA, RHOU, CXCL8, SEPT6, EPGN, RDX, WNT5A,* and *EZH2*) during the culture. The cellular ultrastructure showed the presence of structures characteristic of mitotic cell division: a centrosome surrounded by a pericentric matrix, a microtubule system, and a mitotic spindle connected to chromosomes. The main goal of the study was to identify the genes involved in mitotic division and to identify the cellular ultrastructure of GCs in a long-term in vitro culture. All of the genes in these groups were subjected to downstream analysis, and their function and relation to the ovarian environment are discussed. The obtained results suggest that long-term in vitro cultivation of GCs may lead to their differentiation toward another cell type, including cells with cancer-like characteristics.

## 1. Introduction

In the last phase of preovulatory follicle growth, mature oocytes are accompanied by somatic cells known as granulosa cells (GCs), with facilitation of the final steps of estrogen synthesis being their main function. From an oocyte viewpoint, the most important function of GCs is their participation in bidirectional communication, which allows for the exchange of signals and cellular metabolites between these two cell types [1]. GCs are an important factor in the comprehension of the folliculogenesis process [2,3]. However, understanding their transcriptome may be an important step in expanding knowledge about their possible application to medicine of the 21st century. This includes the significant stem-like potential of in vitro cultured GCs (described by Kossowska-Tomaszczuk et al.), which could be employed in the fields of regenerative and reconstructive medicine [4,5,6,7]. In addition, an analysis of GC gene expression in long-term in vitro cultures could help to define new therapeutic goals in the rapidly expanding field of infertility treatment [8]. 

DNA expression microarrays have become a useful technique in gene expression analysis [9], providing the opportunity to learn about many genetic pathways involved in the processes associated with in vitro culture conditions [10]. Microarray technology has enabled the discovery of many ontological groups involved in molecular cell division processes [11,12]. An analysis of the GC transcriptome during long-term in vitro culture may be useful in expanding knowledge about the mechanisms involved in their proliferation and possible differentiation in conditions different from physiological ones [13]. 

Hence, the major aim of this study was to describe the expression of genes involved in several gene ontologies associated with the process of cellular division (“cell cycle arrest”, “cell cycle process”, “mitotic spindle organization”, “mitotic spindle assembly checkpoint”, “mitotic spindle assembly”, and “mitotic spindle checkpoint”) during a primary long-term in vitro culture. The resulting set of genes of interest were then related to current knowledge about their involvement in physiological and in vitro granulosa-associated processes. This allowed for a better understanding of the molecular drivers underlying the in vitro proliferation of human granulosa cells and determined new markers of that process that had not been previously associated with cell building in the follicle. The obtained data could serve as a basic molecular reference for further studies that aim to fully encompass the mechanisms of in vitro cultured granulosa, ultimately aiming to employ these cells in fields such as assisted reproduction or regenerative medicine. The study additionally employed transmission electron microscopy (TEM) to provide additional information about the cells present in the culture and in cell-cycle-associated processes.

## 2. Materials and Methods 

### 2.1. Patient Selection and Granulosa Cell Separation

Patients subjected to in vitro fertilization (IVF) procedures were the source of GCs in our study. After giving consent, 20 patients with diagnosed infertility (aged 18–40) qualified for this study. Patients with a potential risk of inadequate ovarian stimulation—according to Bologna’s criteria of poor ovarian responders, which was published by the European Society of Human Reproduction and Embryology (ESHRE) in 2011 [14] were excluded (using a serum antimullerian hormone (AMH) level of 0.7 ng/mL as a cut-off value). Moreover, patients with endometriosis, polycystic ovary syndrome (PCOS), fewer than 9 antral follicles, and/or day 2–3 follicle-stimulating hormone (FSH) serum levels higher than 15 mU/mL were excluded. Furthermore, each of the samples included in the study was collected from a patient who had had 2–5 ovarian follicles punctured during the procedure to improve the reliability of the results. In vitro fertilization procedures were conducted in the Department of Infertility and Reproductive Endocrinology, Poznań Medical University, Poznań.

Patients with previously diagnosed infertility were subjected to the procedure of controlled ovarian hyperstimulation. For ovarian stimulation, FSH (Gonal-F, Merck Serono, Darmstadt, Germany) and highly purified human menopausal gonadotropin (hMG-HP, Menopur, Ferring, Saint-Prex, Switzerland) were used. To suppress pituitary function, injections of Cetrorelix Acetate (Cetrotide, Merck Serono, Darmstadt, Germany) were administered in an adequate dose. Ovulation was evoked by subcutaneous injection of 6500 U of human chorionic gonadotropin (hCG; Ovitrelle, Merck-Serono, Darmstadt, Germany). After oocyte pick-up (OPU), cumulus–oocyte complex (COC) and the remaining follicular fluid (FF) from each ovary were collected for further analysis. FF with suspended GCs was collected only from ovarian follicles larger than 16 mm. To separate and collect GCs, the FF samples were subjected to centrifugation for 10 min at 200× *g* to obtain a GC-containing pellet [15,16]. The Poznań University of Medical Sciences Bioethical Committee gave approval for the study under resolution 558/17. All patients meeting the assumed criteria of the study expressed their informed, written consent for participation in this research.

### 2.2. Cell Culture

The cells obtained from patients undergoing IVF fertilization procedures were washed twice in culture medium through centrifugation at 200× *g* for 10 min at room temperature (RT). The culture medium consisted of Dulbecco’s Modified Eagle Medium (DMEM, Sigma; Merck KGaA, Darmstadt, Germany), 2% fetal bovine serum (FBS, Sigma; Merck KGaA, Darmstadt, Germany), 10 mg/mL gentamicin (Invitrogen; Thermo Fisher Scientific, Inc., Waltham, MA, USA), 4 mM L-glutamine (stock 200 mM, Invitrogen; Thermo Fisher Scientific, Inc., Waltham, MA, USA), 10,000 μg/mL streptomycin, and 10,000 U/mL penicillin (Invitrogen; Thermo Fisher Scientific, Inc., Waltham, MA, USA) [15,17].

The GC culture was maintained in conditions of 37 °C and 5% CO_2_. When it reached 90% confluency, the cells were separated from the vessel using 0.05% trypsin- EthylenoDiamineTetraAcetic (trypsine – EDTA Invitrogen; Thermo Fisher Scientific, Inc., Waltham, MA, USA) for 1–2 min and were subjected to counting with the use of an ADAM Cell Counter and Viability Analyzer (ADAM CCVA) (Bulldog Bio, Portsmouth, NH, USA) (Adam CCVA). The culture was carried out for 30 days. The medium was changed twice per week. Total RNA was isolated after 1, 7, 15, and 30 days of culture. The ADAM CCVA was used to test the viability of each collected sample, and samples containing 95% or more viable cells were used for subsequent molecular analyses. The samples from each patient were cultured separately, with the RNA material from specific culture periods pooled before the microarray and RT-qPCR analysis. Different time periods were obtained from different patient samples, with pooling serving to alleviate the patient-specific transcript variation.

### 2.3. Transmission Electron Microscopy Analysis

For the transmission electron microscopy analysis, a suspension containing a fraction of human ovarian granulosa cells harvested during the mentioned time intervals of in vitro primary culture was used. The cells were fixed in 2.5% glutaraldehyde (Polysciences, Inc., Hirschberg an der Bergstraße, Germany) and rinsed with phosphate buffer. Next, the samples were postfixed with 1% osmium tetroxide. After rinsing the samples in phosphate buffer, dehydration with increasing concentrations of ethanol (from 50% to 100%) was performed at 4 °C. In the next step, the cell pellets were placed in a mixture of ethanol with acetone, acetone, and then an acetone/resin mixture. Later, the samples were embedded in epoxy resin and sliced into thin and ultrathin slices using a Leica Ultracut UCT (Leica Microsystems, Nussloch, Germany). The slices were first stained with toluidine blue and then with uranyl acetate and lead citrate. An ultrastructural analysis of sections considered representative (cells not mechanically damaged) was performed using a JEM 1010 (Jeol, Tokyo, Japan).

### 2.4. RNA Isolation

Total RNA was isolated from the GCs of 20 patients after 1, 7, 15, and 30 days of culture using the Chomczyński–Sacchi method [18]. The harvested cells were suspended in 1 ml of phenol and guanidine thiocyanate monophase solution (TRI Reagent^®^, Sigma-Aldrich, St. Luis, MO, USA). To obtain three separate phases, chloroform was added. The RNA was located in the uppermost aqueous phase. Then, the RNA was stripped with 2-propanol (Sigma; Merck KGaA St. Luis, MO, USA; catalog number I9516) and washed with 75% ethanol. Samples of extracted RNA were used for further analysis. The total mRNA amount was determined from the optical density at 260 nm, and the RNA purity was estimated using a 260/280-nm absorption ratio (NanoDrop spectrophotometer, Thermo Scientific, Poland). Samples with a 260/280 absorbance ratio greater than 1.8 were used in the study.

### 2.5. Microarray Analysis

The microarray analysis was conducted according to protocols used in previous studies by our team [16,19,20,21]: 100 ng RNA from each pooled sample (2 samples from each time interval) was subjected to two rounds of sense cDNA amplification (Ambion^®^ WT Expression Kit, Ambion, Austin, TX, USA). The resulting cDNA was biotin-labeled and further fragmented using an Affymetrix GeneChip^®^ WT (Affymetrix, Santa Clara, CA, USA). The biotin-labeled (5.5 μg) fragments were hybridized with an Affymetrix^®^ U219 Human Genome Array (Affymetrix, Santa Clara, CA, USA) (48 °C/20 h). Then, they were washed and stained with the use of an Affymetrix GeneAtlas Fluidics station. The array stripes were then subjected to scanning using the GeneAtlas system imaging station. Affymetrix GeneAtlas^TM^ Operating Software (v. 2.0.0.460, Affymetrix, Santa Clara, CA, USA) was used for the reading analysis. Data quality was approved in accordance with the software quality control criteria. The obtained CEL files were subjected to further data analysis [22,23].

Bioconductor software (v. 3.1.0, www.bioconductor.org) and R 3.5.1 programming language (www.r-project.org) were used for the analysis and graph compilation. The CEL files were merged with the description file. The background was corrected with the use of robust multiarray averaging (RMA), which was also employed for results normalization and summarization. The differentially expressed genes were selected on the basis of an adjusted *p*-value lower than 0.05 and an expression greater than two-fold. A list of differentially expressed genes (separated for up- and downregulated genes) was uploaded to DAVID software (v.6.8, Database for Annotation, Visualization and Integrated Discovery; Leidos Biomedical Research, Inc., National Cancer Institute, Frederick, MD, USA) (a database for annotation, visualization, and integrated detection) [24].

To predict the interactions between individual genes, sets of genes belonging to the Gene Ontology Biological Processes (GO BPs) of interest were uploaded into the STRING software (v. 10, Search Tool for Retrieval of Interacting Genes/Proteins, STRING Consortium, Lausanne, Switzerland) [25]. The STRING database contains data on protein/gene interactions that have been obtained through experimentation, computational forecasting methods, and analyses of the available public literature.

In order to further investigate the selected sets of genes, we examined their mutual relations with the GOplot package [26].

### 2.6. RT-qPCR Analysis

To confirm the results obtained from the microarrays, RT-qPCR was performed: 10 upregulated genes and 10 downregulated genes were selected from the GOs of interest. Three biological samples of each gene were used for the analysis. Each analysis was carried out in three technical repetitions. Reverse transcription was performed using SABiosciences (RT2 First Stand Kit-330401) and a 96-well Veritimer thermocycler. For the reverse transcription, 1 mg of RNA transcript was used [17].

Real-time PCR was performed using a Light Cycler^®^ 96 (Roche Diagnostic GmbH, Manheim, Germany), RT2 SYBR^®^ Green ROX™ qPCR Master Mix (Qiagen Sciences, Gaithersburg, MD, USA), and sequence-specific primers (Table 1).

For the controls, 3-phosphate glyceraldehyde dehydrogenase (GADPH), β-actin (ACTB), and hypoxanthine 1 (HPRT1) phosphoribosyl transferase were used. The gene expression was analyzed using relative quantification (RQ). The RT-qPCR primers were designed using Primer3Plus software (version 0.4.0; Whitehead Institute for Biomedical Research, Massachusetts Institute of Technology, Cambridge, MA, USA).

### 2.7. Statistics

Bioconductor software (v. 3.1.0, www.bioconductor.org) and R 3.5.1 programming language (www.r-project.org) served as tools to perform the statistical analyses. The empirical Bayes method was used to obtain the adjusted *p*-value of all the genes of interest, with Benjamini and Hochberg’s false discovery rate used to correct the results for multiple comparisons (*p* < 0.05 indicated a statistically significant difference). The same methods and selection criteria were used to estimate the statistical significance of the enriched GO terms and Kyoto Encyclopedia of Genes and Genomes (KEGG) pathways, with DAVID database software (v.6.8, Leidos Biomedical Research, Inc., National Laboratory for Cancer Research, Frederick, MD, USA; david.ncifcrf.gov) employed for these analyses. Each GO term and KEGG pathway was considered significantly enriched if it contained at least 5 differentially expressed genes, with *p* < 0.05. An RT-qPCR statistical analysis was performed using the Real Statistics Resource Pack for MS Excel 2016, also using the abovementioned methods (Microsoft Corporation, Redmond, WA, USA) [15,27,28].

## 3. Results

In this study, Affymetrix microarrays were used to determine the changes in expression of the GC transcriptome between the 1st, 7th, 15th, and 30th day of in vitro culture. An Affymetrix^®^ Human HgU 219 Array analysis yielded data about the expression of 22,480 transcripts. To be considered differentially expressed, the genes needed to exceed a fold change of |2| and be characterized with a *p*-value below 0.05. The inclusion criteria resulted in the qualification of 2278 different transcripts for the study. The complete set of genes detected in the microarray analysis was uploaded to the GEO database (https://www.ncbi.nlm.nih.gov/geo/query/acc.cgi?acc=GSE129919).

Here, 218 differentially expressed genes could be found in the GO BPs of interest. From that set, the genes of the 10 highest and 10 lowest mean changes in expression between 7, 15, and 30 days of culture were chosen for subsequent analyses.

**Figure 1:**
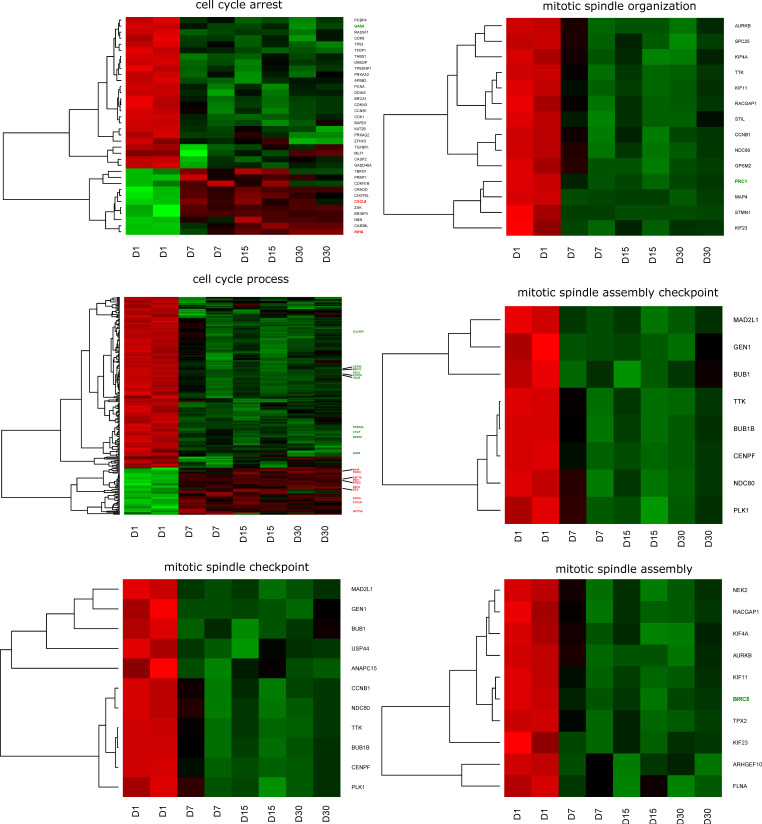
Heat map representation of differentially expressed genes belonging to the “cell cycle arrest”, “cell cycle process”, “mitotic spindle assembly checkpoint”, “mitotic spindle assembly”, “mitotic spindle checkpoint”, and “mitotic spindle organization” gene ontology biological process (GO BP) terms. The arbitrary signal intensity acquired from the microarray analysis is represented by colors (green is higher expression and red is lower expression). Log_2_ signal intensity values for any single gene were resized to a row *z*-score scale (from -2, the lowest expression, to +2, the highest expression for a single gene).

Genes belonging to the “cell cycle arrest”, “cell cycle process”, “mitotic spindle assembly checkpoint”, “mitotic spindle assembly”, “mitotic spindle checkpoint”, and “mitotic spindle organization” gene ontologies (gene ontology biological process terms (GO BPs)) were extracted with the use of DAVID software (Database for Annotation, Visualization, and Integrated Discovery). The DAVID analysis was applied separately to up- and downregulated gene sets, with an adjusted *p*-value below 0.05 serving as a criterion for selection. This allowed for the identification of 582 GOs and 45 KEGG pathways that contained differentially expressed genes. A hierarchical clustering procedure was applied to the sets of genes of interest, which allowed for presenting the results in the form of heatmaps (Figure 1). Additionally, the exact fold expression changes of each of the analyzed genes are presented in Table 2.

**Figure 2:**
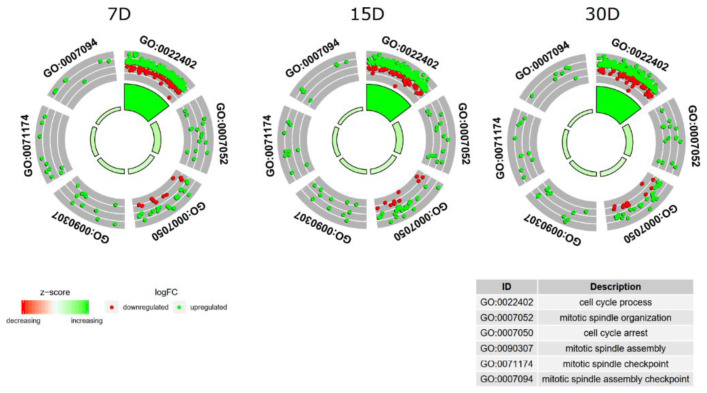
A circular visualization of the results of the gene annotation enrichment analysis. The outer circle shows a scatterplot for each term of the fold change logarithm (logFC) of the assigned genes. Green circles display upregulation, and red ones display downregulation. The inner circle is the representation of the *z*-score. The size and the color of the bar correspond to the value of the *z*-score. D-days of culture.

Measurement of the enrichment levels of each of the GO BPs of interest was then used for further investigation of the changes in expression of the genes that they contained. These values were visualized as a circle in the form of *z*-scores (Figure 2).

**Figure 3:**
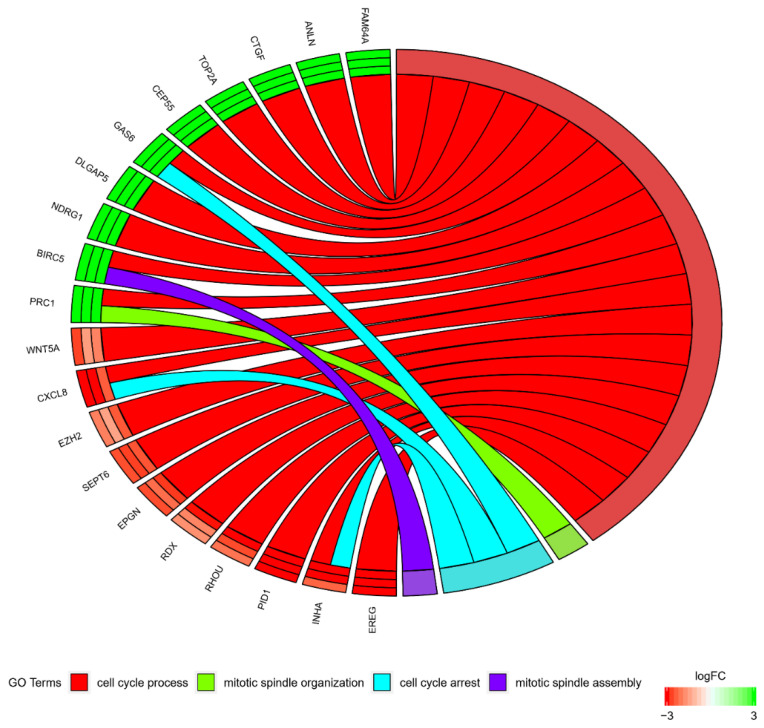
A representation of the mutual relationship between the 20 chosen genes that belonged to the “cell cycle arrest”, “cell cycle process”, “mitotic spindle assembly checkpoint”, “mitotic spindle assembly”, “mitotic spindle checkpoint”, and “mitotic spindle organization” GO BP terms. The ribbons indicate which gene belongs to which category. The colors of the three inner bars near each gene correspond to the logFC after 7, 15, and 30 days. The genes were sorted by logFC.

At the same time, most of the genes did not exclusively belong to a singular GO. Because of that, the intersections between the GOs of interest were analyzed and are presented in a circle diagram (Figure 3).

**Figure 4:**
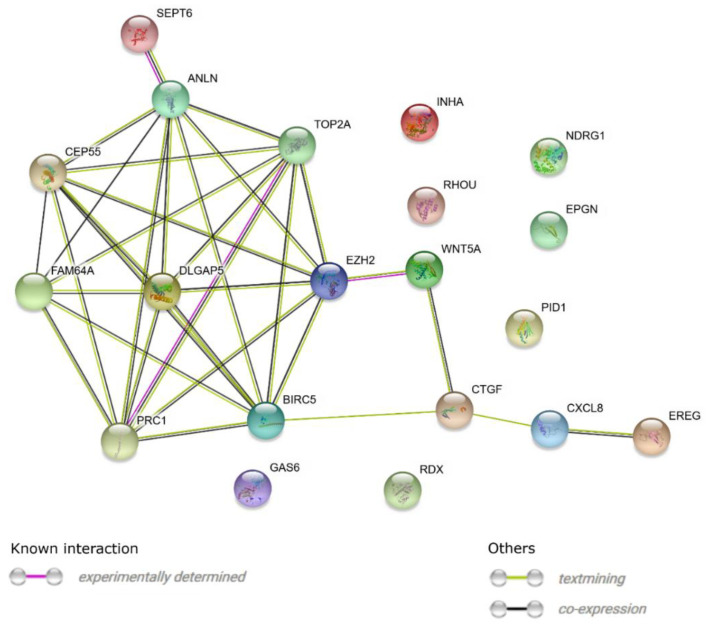
STRING-generated interaction network between the 20 chosen genes belonging to the “cell cycle arrest”, “cell cycle process”, “mitotic spindle assembly checkpoint”, “mitotic spindle assembly”, “mitotic spindle checkpoint”, and “mitotic spindle organization” GO BP terms. The intensity of the edges reflects the strength of the interaction score.

In addition, STRING software was used to study the molecular interaction networks between the 20 selected genes. The detected networks included experimentally determined interactions, co-expression, and interactions determined through text-mining (Figure 4).

**Figure 5:**
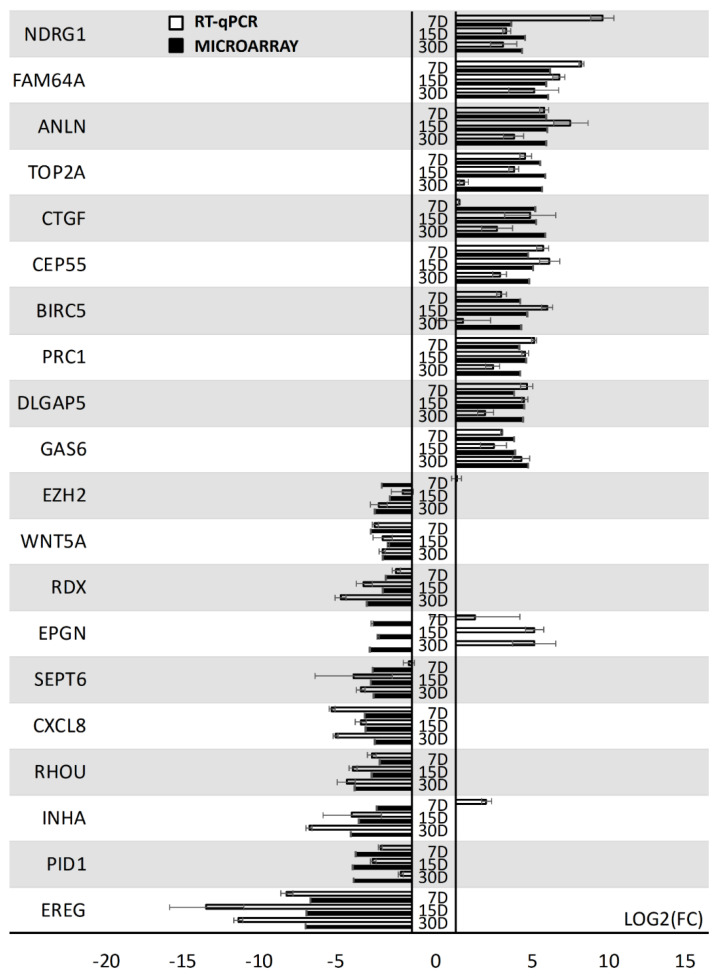
The results of the RT-qPCR validation of expression microarrays (log (FC)), presented as a bar graph. Error bars represent the standard error of the mean (SEM; *n* = 3). All of the presented sample means were deemed to be statistically significant (*p* < 0.05). D: day of culture; FC: fold change.

To quantitatively validate the results of the microarray analysis, the RT-qPCR method was employed. The results of the validation are presented as a bar graph (Figure 5).

The analysis validated the course of expression in the majority of the 20 selected genes. Only one gene showed an opposite change in expression in one of the analyzed time periods (*EPGN*). The resulting variable result may have been caused by the fact that expression microarrays are a wide-scale method, while RT-qPCR was used to detect particular transcript variants with much larger quantitative precision. As fold changes are highly dependent on the values of individual sample expression levels, the difference in sensitivity of the methods can sometimes cause discrepancies [29]. In such cases, RT-qPCR results are usually treated as more representative, which was also the approach used in this study.

**Figure 6:**
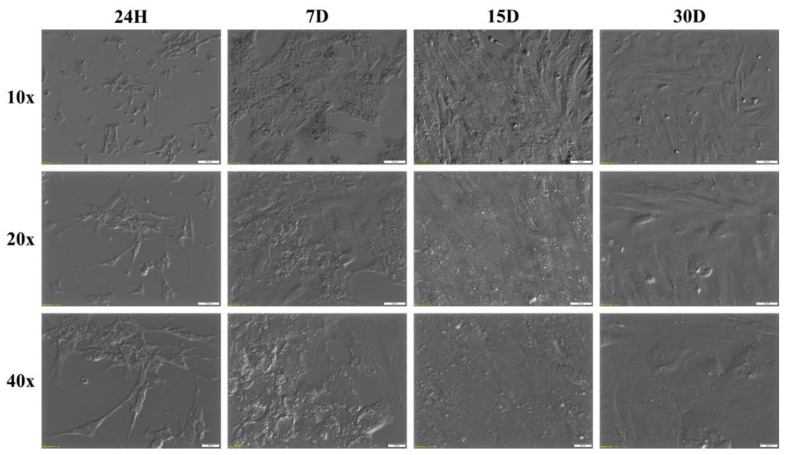
Change in granulosa cell (GC) morphology during long-term in vitro primary culture. 24H: first day of culture; D: day of culture; 10×, 20×, 40×: magnification.

**Figure 7:**
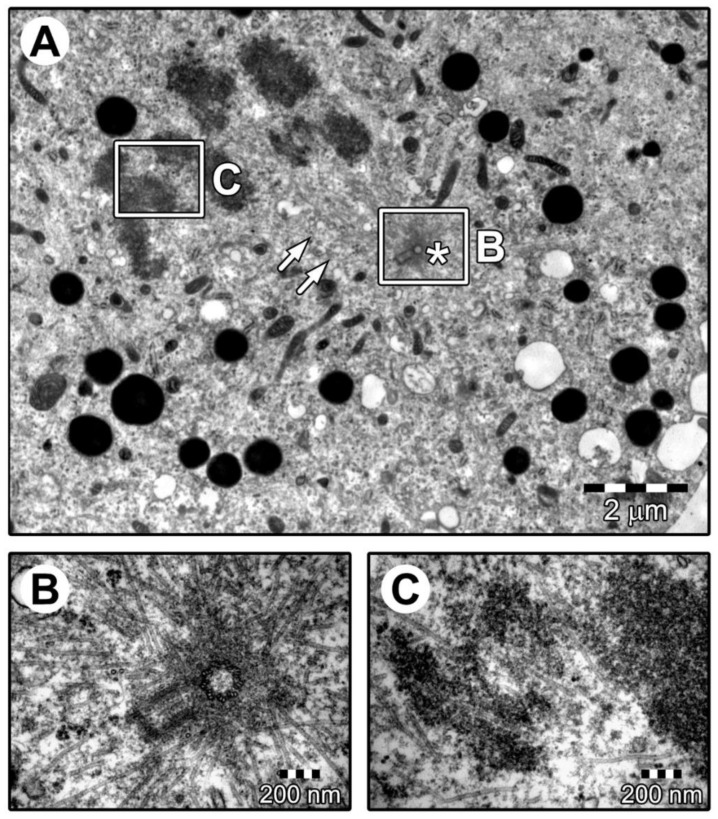
Human ovarian granulosa cells during mitotic division. Human ovarian GC cultures (day 7). Micro-electrophotography showing cells during mitotic division (**A**). The centrosome (star-like shape) is surrounded by a cloud of amorphous material forming a pericentric matrix, along with a radial microtubule system (**B**). Structures of the mitotic spindles (arrows) are visible, connecting to the chromosomes located in the equatorial plane (**C**).

To further investigate the potential phenotypical changes of the cells of interest, a morphological and ultrastructural evaluation was performed using light and transmission electron microscopy. The morphology of the GCs changed from a star-like cell shape into an elongated, fibroblast-like cell shape. Figure 6 shows the morphology of GCs at individual time intervals of the in vitro primary culture. During the analysis of cellular ultrastructure, the focus was placed primarily on structures related to mitotic cell division. The analysis showed the presence of a centrosome surrounded by a cloud of amorphous material forming a pericentric matrix with a radial microtubule system. Another characteristic structure for this process was mitotic spindles. The mitotic division of human GCs is illustrated in Figure 7.

## 4. Discussion

In this study, an analysis of GC transcriptomes in individual time intervals was supplemented by an examination of their ultrastructure. The obtained results clearly indicate that these cells proliferated in long-term primary in vitro culture. This was indicated by the presence of centrosomes surrounded by the pericentric matrix, as well as the radial microtubule system. In addition, the mitotic spindle connected with chromosomes located in the equatorial plane. Earlier analyses using an electron transmission microscope focused primarily on structures related to the secretory properties of these cells [30,31]. Lipid droplets were also present in the cells (not marked in the figure). The gathering of lipid droplets in the cytoplasm of GCs confirmed the presence of these cells in the in vitro primary culture and testified to their metabolic activity. In many of the studies conducted on GCs, it has been suggested that the secretory properties of GCs in primary culture can be used in a coculture with embryos [32]. The research presented here suggests that GCs are capable of proliferation in in vitro culture conditions.

A transcriptomic analysis of proliferating GCs was the main goal of this study. Human GCs undergoing mitotic division showed differentially expressed genes belonging to the “cell cycle arrest”, “cell cycle process”, “mitotic spindle organization”, “mitotic spindle assembly checkpoint”, “mitotic spindle assembly”, and “mitotic spindle checkpoint” GOs during long-term primary culture conducted in in vitro conditions. The obtained results indicate the key role of these genes during granulosa cell division processes. In our research, special attention was paid to two groups of genes. The first of them included genes belonging to “cell cycle arrest” and “cell cycle process”, which are responsible for the regulation of the cell cycle by restricting the progression of the cell cycle during one of its four phases: G1, S, G2, or M. The second class of genes, which contained members of the other four analyzed ontological groups, consisted of genes taking part in the organization and arrangement of the karyokinetic spindle structure during mitosis. During our study, among the members of the gene ontologies of interest, we chose the 10 most upregulated and 10 most downregulated genes. In the course of conducting the analyses, it was found that an increase in gene expression was particularly observed in the “cell cycle arrest” and “cell cycle process” ontology groups. The genes with the most increased expression were *FAM64A, ANLN, TOP2A, CTGF, CEP55, BIRC5, PRC1, DLGAP5, GAS6,* and *NDRG1*; while *EREG, PID1, INHA, RHOU, CXCL8, SEPT6, EPGN, RDX, WNT5A,* and *EZH2* were the most downregulated during the time of the culture. While most of the microarray results were validated using RT-qPCR, it needs to be noted that *EPGN* showed an opposite pattern of expression across all of the culture periods, while *INHA* exhibited a reversed expression pattern in the first analyzed period of the culture. In these cases, as RT-qPCR is considered to be more quantitatively accurate, the results of the validation were considered in the discussion. The interactions and relations of these genes are presented in Figure 2, Figure 3, and Figure 5. Some of the genes of interest represented more than one ontology groups (Figure 4). All of them belonged to the “cell cycle process” ontology group.

The most upregulated gene was *FAM64A* (*family with sequence similarity 64 member A*), which is also called *CATS*. During cell division, *CATS* controls the transition from the metaphase to the anaphase and is considered to be a proliferation marker. A strong expression of *CATS* is observed in cancer cell lines. Depending on the cell cycle stage, it is induced by mitogens [33]. This gene has been demonstrated to belong to the group of three genes with the highest level in more aggressive *triple-negative breast cancer* (TNBC) [34]. A high expression of this gene in granulosa cells may reflect a high potential for proliferation and differentiation resembling that occurring in the aggressive forms of breast cancer. The next most upregulated gene was *ANLN* (*anillin actin-binding protein*), which encodes an actin-binding protein that is important for growth, migration, and cell cytokinesis [35]. *ANLN* coordinates and is responsible for the synthesis of several key factors involved in cell division during cytokinesis. Its important roles also involve extracellular adhesion control in mammalian epithelial cells through JNK (c-Jun N-terminal kinase) activity suppression and gap junction regulation [36]. Like with *CATS*, elevated *ANLN* expression has been found in many human cancers, including in breast, endometrial, and ovarian cancers [37]. *TOP2A* (*DNA topoisomerase II alpha*) controls the state of DNA during transcription and plays an essential role in DNA stabilization. The enzyme encoded by this gene coordinates chromosomal condensation processes and chromatid formation and relieves any mechanical strains occurring during transcription and DNA replication. *TOP2A* can play an important prognostic role in epithelial ovarian cancer (EOC) [38]. It has also been indicated that *TOP2A* overexpression occurs in ovarian tumors: it is currently the main target of clinical trials of ovarian cancer therapies [39]. The next analyzed gene was *CTGF* (*connective tissue growth factor*), which is often referred to as *CCN2*. It encodes a protein that is secreted mainly during various pathological tissue conditions, such as increased fibrogenesis and tissue fibrosis. The expression of this gene has been found in several cases of tumors. Members of the connective tissue growth factor (CCN) family regulate cell migration, proliferation, apoptosis, differentiation, and extracellular matrix remodeling. In females, it plays an important role in reproductive organ regulation. Animal models have proven that *CTGF* is overexpressed in granulosa cells during ovarian follicular development [40]. The current results combined with the results of Cheng et al. [41] confirm the results presented in this study. *CEP55* (*centrosomal protein 55*) is responsible for cytokinesis, and its overexpression is associated with a genetic instability characteristic of breast cancers [42]. The next gene, *BIRC5* (*baculoviral IAP repeat containing 5*), represented the “mitotic spindle assembly” and “cell cycle process” ontology groups. This gene has two basic functions: cell death regulation and mitosis progression control. As in the case of the previously discussed genes, increased *BIRC5* overexpression characterizes most types of cancers [43]. The last four most upregulated genes were *PRC1* (*protein regulator of cytokinesis 1*), *DLGAP5* (*DLG-associated protein 5*), *GAS6* (*growth arrest specific 6*), and *NDRG1* (N-myc downstream regulated 1). All four genes are overexpressed in various tumors and are considered to be prognostic indicators for several types of cancer. Interestingly, *PRC1* is downregulated in Mtor-ZcKO oocytes. Taking the role of PRC1 (*protein regulating cytokinesis 1)* in the control of somatic cell cytokinesis division into account, a reduced level of its expression in oocytes may indicate that they have reached maturity [44]. From the group of 10 selected downregulated genes, the expression of *EREG* (*epiregulin*) decreased the most. Expression of the *EREG* gene was found in the ovarian GCs as well as in liver progenitor cells. Epiregulin belongs to the EGF (epidermal growth factor) family. It has been proven that its main role is its participation in oocyte maturation, ovulation promotion, and subsequent embryo implantation [45]. A decrease in this gene’s expression in the primary in vitro GC culture may have resulted from a lack of bidirectional communication with the oocyte (occurring in physiological conditions). *PID1 (phosphotyrosine interaction domain-containing 1)* belongs to the family of AKT signaling proteins, additionally mediating antilipolytic insulin action through AKT pathway transduction [46]. The role that it can play in ovarian tissue has not yet been recognized. The next most downregulated gene, representing the “cell cycle process” and “cell cycle arrest” ontology groups, was *INHA* (inhibin subunit alpha). However, it needs to be noted that its expression was found to be upregulated on day 7 of the primary culture during RT-qPCR validation. Observed mutations in this gene may result in premature ovarian failure in patients. Protein complexes containing *INHA* negatively regulate the secretion of FSH from the pituitary gland, which may in some way explain the decrease of this gene’s expression during the in vitro GC culture. *RHOU* (ras homolog family member U) mediates the WNT1 signaling pathway, also affecting morphology, proliferation, and cytoskeleton organization [47]. *CXCL8* (C-X-C motif chemokine ligand 8) represents the family of inflammatory response mediators. The overexpression of this gene results in poor prognosis in several solid tumors, including epithelial ovarian cancer (EOC). In ovarian cancer, *CXCL8* expression induction increases cell viability and proliferation and reduces apoptosis [48]. The last five genes classified in the downregulated group belonged only to the “cell cycle process” ontology group. Among these genes, we could observe very similar levels of expression. This group included the *SEPT6* (*septin-6), EPGN (epithelial mitogen), RDX (radixin), WNT5A (Wnt family member 5A), and EZH2 (enhancer of zeste 2 polycomb repressive complex 2 subunit)* genes. Septins belong to the group of cytoskeleton GTP-binding proteins and take part in cytokinesis during cell division [49]. *EPGN* stimulated epidermal cell proliferation in the in vitro culture. As a member of the EGF family, it induces cumulus cell expansion in growing ovarian follicles. Due to its biochemical properties, *EPGN* may be an indispensable factor for maintaining cell signaling, and it was also the only gene in this study with an opposite expression pattern detected during RT-qPCR validation [50]. Radixin belongs to the group of cytoskeletal proteins, while Wnt5a affects cell migration processes and tissue angiogenesis. It also has important functions in tissue repair and the maintenance of their permanent state. In healthy ovaries, Wnt5A expression is significantly higher than in those with diagnosed cancer. Wnt5a expression significantly inhibits the proliferation of human EOC cells [51]. *EZH2* has oncogenic activity. Its inhibition (*EZH2*) may disturb cell signaling pathways that lead to tumor cell proliferation, and may simultaneously promote apoptosis in these cells [52].

## 5. Conclusions

The current literature and the results for the genes identified in this study may be used to describe the processes of human ovarian granulosa cells associated with the regulation of cell division and mitotic spindle formation in vitro. The current data showed the differential expression of genes involved in human granulosa cell proliferation processes. GC gene expression was evaluated for up to 30 days of in vitro primary culture. From the six ontological groups (cell cycle arrest (GO: 0007050), cell cycle process (GO: 0022402), mitotic spindle organization (GO: 0007052), mitotic spindle assembly checkpoint (GO: 0007094), mitotic spindle assembly (GO: 0090307), and mitotic spindle checkpoint (GO: 0071174)) we selected and described the 10 most down- and 10 most upregulated genes. Interestingly, most of the analyzed genes were characteristic for the carcinogenesis process. Moreover, the gene overexpression profile in the group of upregulated genes corresponded with the increased expression of these genes in tumor cells. The similar expression profile and ultrastructural imaging between differentiated and divided human GCs in primary culture may indicate their potential to differentiate into cells with cancer-like characteristics. Most of the analyzed genes are used in cancer-related therapeutic approaches. Our results may contribute to the identification of new genes that are helpful in conducting ovarian carcinoma therapies. Moreover, the current findings may indicate new morphological markers for the granulosa cell mitotic division mechanism in growing ovarian follicles. The identification of key genes involved in this process could be explored in subsequent studies. Above all, this study connects a range of currently known cell-cycle-associated genes to in vitro cultured ovarian granulosa cells, bringing further insight into the potential effects of long-term in vitro cultures on the ex vivo behavior of human GCs.

## Figures and Tables

**Table 1:** Oligonucleotide sequences of primers used for RT-qPCR analysis: the primers of the 10 most upregulated and 10 most downregulated genes.

Gene Name	Primer Sequence (5′–3′)	Product Size (bp)
*NDRG1*	F: ACAACCCTGAGATGGTGGAG R: TGTGGACCACTTCCACGTTA	174
*GAS6*	F: ATCAAGGTCAACAGGGATGC R: CTTCTCCGTTCAGCCAGTTC	187
*DLGAP5*	F: CCAGTCGACACAGGAAGGAT R: CATTGCCCTTGGCTTAACAT	227
*PRC1*	F: TAGACCACACCCCAGACACA R: GTGGCCACAGCTTCTCTTTC	223
*BIRC5*	F: GCCTTTCCTTAAAGGCCATC R: AACCCTTCCCAGACTCCACT	187
*CEP55*	F: CAGTATCCAGCCACTGAGCA R: GGGAGGTATCACTGCCAAGA	245
*CTGF*	F: GGAAAAGATTCCCACCCAAT R: TGCTCCTAAAGCCACACCTT	153
*TOP2A*	F: AATCTCAGAGCTTCCCGTCA R: TGCCTCTGCCAGTTTTTCTT	175
*ANLN*	F: ATGCAGTGTGGTGCACATTT R: AACCCAAACACTTTGGCAAG	195
*FAM64A*	F: GGAGATCTCTCCAGCACCAG R: GCACCCAAAGCACTCTTAGC	244
*EZH2*	F: AGGACGGCTCCTCTAACCAT R: CTTGGTGTTGCACTGTGCTT	179
*WNT5A*	F: TGGCTTTGGCCATATTTTTC R: CCGATGTACTGCATGTGGTC	199
*RDX*	F: CCCAGAGACTCTTCTTCTTGC R: TACACGCTGGGGTAGGAGTC	179
*EPGN*	F: TGACAGCACTGACCGAAGAG R: CTCATGGTGGAATGCACAAG	188
*SEPT6*	F: TCTGCTTCAACATCCTGTGC R: GCTTTAGCCTCACGTTGCTC	168
*CXCL8*	F: AAGAAACCACCGGAAGGAAC R: AAATTTGGGGTGGAAAGGTT	183
*RHOU*	F: AGGCCTCTCTGCTACACCAA R: TCAGGCACTGGCTTTTCTTT	215
*INHA*	F: CCAGCTGTGAGGACAAGTCA R: CTAGCAGGGGCTCAGAGCTA	186
*PID1*	F: TACCTGGGCAAAGTCTCCAC R: TTTGTGGTCGAGATGATGGA	171
*EREG*	F: CCAAGGACGGAAAATGCTTA R: AAAATTAGCTGGGCATGGTG	237
*GAPDH*	F: TCAGCCGCATCTTCTTTTGC R: ACGACCAAATCCGTTGACTC	90
*ACTB*	F: AAAGACCTGTACGCCAACAC R: CTCAGGAGGAGCAATGATCTTG	132
*HPRT1*	F: TGGCGTCGTGATTAGTGATG R: ACATCTCGAGCAAGACGTTC	141

F: forward primer; R: reverse primer.

**Table 2:** Gene symbols, fold changes in expression, Entrez gene IDs, and adjusted *p*-values of the studied genes. Red: downregulated genes; green: upregulated genes.

Gene Symbol	Fold Change D7/D1	Fold Change D15/D1	Fold Change D30/D1	Adjusted *p*-Value D7/D1	Adjusted *p*-Value D15/D1	Adjusted *p*-Value D30/D1	Entrez Gene ID
*EREG*	0.010	0.008	0.008	0.0002	0.0002	0.0002	2069
*PID1*	0.076	0.068	0.071	0.0024	0.0020	0.0017	55022
*INHA*	0.204	0.090	0.063	0.0266	0.0066	0.0036	3623
*RHOU*	0.236	0.167	0.073	0.0481	0.0226	0.0061	58480
*CXCL8*	0.116	0.122	0.191	0.0189	0.0180	0.0337	3576
*SEPT6*	0.172	0.156	0.179	0.0006	0.0005	0.0005	23157
*EPGN*	0.169	0.220	0.149	0.0437	0.0621	0.0289	255324
*RDX*	0.306	0.273	0.129	0.0227	0.0153	0.0033	5962
*WNT5A*	0.158	0.347	0.268	0.0184	0.0825	0.0407	7474
*EZH2*	0.259	0.369	0.185	0.0016	0.0031	0.0006	2146
*NDRG1*	11.930	22.621	20.319	0.0022	0.0011	0.0009	10397
*GAS6*	13.923	14.854	26.709	0.0059	0.0051	0.0024	2621
*DLGAP5*	14.126	22.518	20.818	0.0230	0.0124	0.0120	9787
*PRC1*	18.098	23.764	18.552	0.0016	0.0012	0.0012	9055
*BIRC5*	18.745	25.651	18.849	0.0029	0.0022	0.0023	332
*CEP55*	26.885	33.896	27.873	0.0056	0.0041	0.0042	55165
*CTGF*	36.067	38.456	56.198	0.0028	0.0025	0.0015	1490
*TOP2A*	45.408	58.461	50.754	0.0026	0.0020	0.0018	7153
*ANLN*	59.052	63.936	59.669	0.0016	0.0013	0.0011	54443
*FAM64A*	71.846	58.770	65.148	0.0009	0.0009	0.0007	54478
